# On the Feasibility of Using Behavioral Listening Effort Test Methods to Evaluate Auditory Performance in Cochlear Implant Users

**DOI:** 10.1177/23312165241240572

**Published:** 2024-04-26

**Authors:** Maartje M. E. Hendrikse, Gertjan Dingemanse, André Goedegebure

**Affiliations:** Department of Otorhinolaryngology and Head and Neck Surgery, Erasmus MC University Medical Center, Rotterdam, the Netherlands

**Keywords:** speech intelligibility, dual task, test–retest reliability, clinical use

## Abstract

Realistic outcome measures that reflect everyday hearing challenges are needed to assess hearing aid and cochlear implant (CI) fitting. Literature suggests that listening effort measures may be more sensitive to differences between hearing-device settings than established speech intelligibility measures when speech intelligibility is near maximum. Which method provides the most effective measurement of listening effort for this purpose is currently unclear. This study aimed to investigate the feasibility of two tests for measuring changes in listening effort in CI users due to signal-to-noise ratio (SNR) differences, as would arise from different hearing-device settings. By comparing the effect size of SNR differences on listening effort measures with test–retest differences, the study evaluated the suitability of these tests for clinical use. Nineteen CI users underwent two listening effort tests at two SNRs (+4 and +8 dB relative to individuals’ 50% speech perception threshold). We employed dual-task paradigms—a sentence-final word identification and recall test (SWIRT) and a sentence verification test (SVT)—to assess listening effort at these two SNRs. Our results show a significant difference in listening effort between the SNRs for both test methods, although the effect size was comparable to the test–retest difference, and the sensitivity was not superior to speech intelligibility measures. Thus, the implementations of SVT and SWIRT used in this study are not suitable for clinical use to measure listening effort differences of this magnitude in individual CI users. However, they can be used in research involving CI users to analyze group data.

## Introduction

Currently, hearing loss and aided performance are typically defined by pure-tone audiometry and speech intelligibility tests in the clinic. While these objective measures can be very sensitive in measuring specific hearing functions, they have limited ecological validity because they use strictly controlled stimuli that are different from the sounds and speech encountered in everyday life. They also have only a moderate correlation with self-perceived handicap (e.g., Chang et al., 2009). Thus, pure-tone thresholds and speech intelligibility measures appear to be limited in predicting the impact of hearing loss on a person's daily activities (Granberg et al., 2014). In addition, speech intelligibility tests are not particularly sensitive to differences in gain-frequency response shape for different hearing-aid settings (Smeds et al., 2019).

In the study by Granberg et al. (2014), many participants reported that it required significant effort to listen and simultaneously perform necessary mental functions involved in communication, such as concentrating, shifting attention, and dividing attention between stimuli. Quantifying this effort required for listening is another approach to evaluating hearing impairment and aided performance, and has received considerable attention in the scientific community in recent years. In the scientific literature, listening effort is defined as *the deliberate allocation of cognitive resources to overcome obstacles or challenges to achieving listening-oriented goals* (Francis & Love, 2020; Pichora-Fuller et al., 2016). Hearing impairment is known to be one of those obstacles, such that hearing-impaired listeners must allocate more cognitive resources (selective attention, working memory) to make sense of a signal in a complex acoustic environment than normal-hearing (NH) listeners (Shinn-Cunningham & Best, 2008). Moreover, cognitive resources are known to have a limited capacity (Kahneman, 1973), so when listening is more effortful, fewer cognitive resources are available for higher-level processing of auditory information (Lunner et al., 2020). Francis and Love (2020) distinguish between the effort demanded by the listening situation (*demanded effort*), the effort actually exerted by the listener in that situation (*exerted effort*), and the listener's perception of how much effort is being expended (*assessed effort*). A measure of exerted listening effort may be a better or complementary indicator of aided performance in challenging situations than speech intelligibility because it reflects the use of cognitive resources needed to perform a task, rather than just task performance. In addition, listening effort measures may be more sensitive at high intelligibility levels, where speech intelligibility measures are less sensitive, due to the different amount of cognitive resources needed to achieve similar intelligibility. Timmer et al. (2018) showed that when listeners with mild hearing loss were provided with hearing aids, this had a greater effect on their listening effort ratings than on their speech understanding ratings. The same may be true for listeners with more severe hearing loss when provided with new hearing devices or hearing-device settings. Therefore, listening effort may be a more powerful measure for evaluating differences between hearing-device settings than speech intelligibility.

Approaches to measuring exerted listening effort can be behavioral methods or physiological. Behavioral methods typically involve a dual-task paradigm. As the primary task requires more listening effort, fewer cognitive resources are available to perform the secondary task and secondary task performance declines. The primary task involves word or sentence recognition, and according to a systematic review by Ohlenforst et al. (2017), the secondary task may use different measures (accuracy, recall, reaction time) and involve different modalities (auditory, visual, tactile). There appears to be no consensus in the literature on which secondary task is best suited for measuring listening effort (Gagne et al., 2017). Finally, examples of physiological methods include functional magnetic resonance imaging, pupillometry, or electroencephalographic response measures during listening task performance (Ohlenforst et al., 2017).

Ohlenforst et al. (2017) concluded that studies investigating whether hearing-aid amplification reduces listening effort have reported mixed results and that there are significant differences in study design and test methods. This observation also holds for studies comparing different hearing-device settings, including different noise reduction algorithms. Desjardins and Doherty (2014) found a significant reduction in listening effort due to noise reduction in hearing-aid users during a dual-task paradigm measuring speech intelligibility and visual tracking. Lunner et al. (2016) and Ng et al. (2013) were able to identify benefits of hearing aid signal processing using a dual-task paradigm measuring speech intelligibility and memory recall for the last word in a sentence. Pals et al. (2020) identified variations in speech comprehension and listening effort among cochlear implant (CI) users with different numbers of active electrodes (7 vs. 15). They employed a dual-task paradigm to assess participants’ accuracy and reaction time in categorizing sentences as true or false. On the other hand, Brons et al. (2013) found no significant reduction in subjective listening effort ratings when comparing different noise reduction algorithms for simulations in NH listeners. Dingemanse and Goedegebure (2022) found no reduction in listening effort with noise reduction algorithm compared to without in CI users using pupillometry. Currently, there is no consensus on the best method for measuring listening effort. An effective method must be sufficiently sensitive to measure differences in listening effort due to different hearing-device settings, and must be applicable to individuals in clinical settings. In order to reach a consensus on which test methods are most appropriate, it is necessary to compare different methods within the same population and under the same conditions.

The current study aimed to investigate the feasibility of using behavioral listening effort tests to measure listening effort in CI users, with the ultimate goal of assessing the feasibility of evaluating different hearing-device settings in the clinic. In addition, a comparison with speech intelligibility measures was made to test the assumption that listening effort measures are more sensitive than speech intelligibility measures at high intelligibility levels. Behavioral listening effort test methods were selected for their suitability in clinical settings, offering ease of administration without requiring specialized equipment. Two behavioral listening effort test methods were compared: a speech recall test (Lunner et al., 2016; Ng et al., 2013), referred to as the sentence-final word identification and recall task (SWIRT); a sentence verification task (SVT) paradigm (Baddeley et al., 1992; May et al., 2001), for which a Dutch corpus is available (Adank & Janse, 2009; Janse & Adank, 2012). These tests have shown promising results in measuring listening effort in hearing-device users (Lunner et al., 2016; Ng et al., 2013; Pals et al., 2020). Both test methods use a dual-task paradigm, providing a measure of speech intelligibility and a measure of listening effort. Focusing on CI users was deliberate, given the greater challenge in discerning differences in listening effort within this group (Perreau et al., 2017). The rationale was that if the chosen test methods proved effective with CI users, they would likely be applicable to hearing-aid users as well. The two behavioral listening effort tests were conducted in two signal-to-noise ratio (SNR) conditions slightly below peak intelligibility performance. To be deemed feasible for clinical use, these tests should be capable of detecting a significant difference in listening effort between the SNR conditions. For clinical utility, the measured difference between the SNR conditions should surpass the test–retest difference, so that conclusions can be drawn based on individual measurements rather than group data and the tests can be used to find the optimal hearing-device setting for an individual. In addition, if the listening effort measures are more sensitive to the SNR difference, we expect the effect of SNR condition to be greater on the listening effort measures than on the speech intelligibility measures when speech intelligibility is near maximum.

## Method

### Participants

CI users (12 male, 7 female) aged between 29 and 79 years (mean: 63.6 years) were included in the study. All participants had a postlingual onset of hearing loss and were implanted more than 6 months before the test. Participants had no known cognitive disabilities and had a phoneme score of at least 65% at 65 dB on clinically used Dutch consonant–vowel–consonant word lists. This should correspond to a word score of >70% for sentences in quiet (Dingemanse & Goedegebure, 2019b), so that a reliable measurement of the 50% speech reception threshold (SRT50) would be possible for sentences in noise (Dingemanse & Goedegebure, 2019a). One participant did not reach 70% intelligibility in quiet for the sentences used in the test and was excluded. The data of the remaining 18 participants were included in the analysis. Participants signed a written informed consent form. The study protocol has been submitted to the Erasmus Medical Center Ethics Committee under the number MEC-2021-0764 and deemed exempt from review.

### SNR Conditions

**Figure 1.**
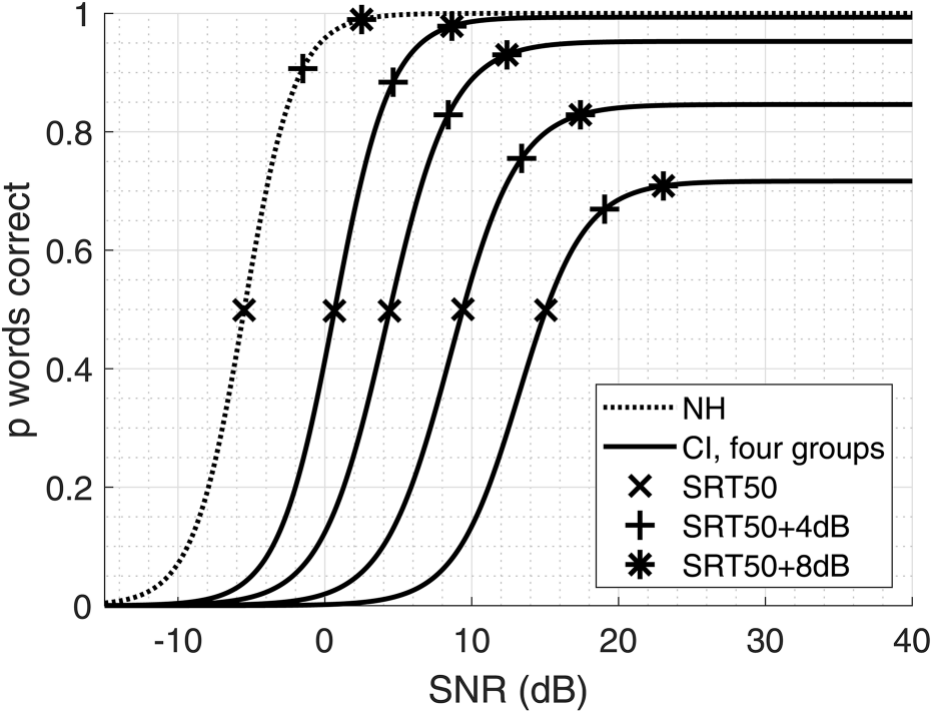
Psychometric curves for NH listeners and CI users were sorted by their SRT50 to create four groups with approximately equally spaced mean SRT50 values, according to Dingemanse and Goedegebure (2020). The SRT50, SRT50 + 4 dB, and SRT50 + 8 dB points are shown to indicate where they lie on the psychometric curve.

In this study, we aimed to select two SNR conditions with a discernible difference in listening effort at high speech intelligibility, and selected SRT50 + 4 dB and SRT50 + 8 dB. Previous studies using SWIRT had based SNR selection on achieving a certain percentage of word recognition from a sentence (85% or 95%; Ng et al., 2013, 2015). For CI users, this approach is not feasible because the intelligibility of the speech material used in the SRT50 measurements varies widely between individuals and does not always reach such high percentages (Dingemanse & Goedegebure, 2020; see Figure 1). To address this challenge, we chose to select SNR conditions relative to each individual's SRT50 measurement, specifically targeting the SNR region where the slope of the psychometric curve flattens (slightly below each individual's maximum intelligibility). In this SNR region speech intelligibility measures are less sensitive than around SRT50, and listening effort measures are believed to be more informative. With this choice of conditions, we can be certain that there is a listening effort difference between conditions.

In addition, we wanted to ensure that the SNR difference reflected a difference that could result from different hearing-device settings. Differences in SNR resulting from different hearing-device settings, such as different noise reduction algorithms, are typically a few dB. The difference between the four algorithms in the study by Brons et al. (2013) was on average 2.5 dB with a maximum of 7 dB for the most effective algorithm. In the current study, a difference of 4 dB between conditions was chosen, which is realistic for an effective noise reduction algorithm.

### SRT50 Measurements

#### Speech Material

SRT50 was measured using the Dutch VU (Vrije Universiteit) sentence test spoken by a native Dutch female speaker (Versfeld et al., 2000). The sentences are unrelated and range in length from five to nine words (median length six words) and were selected from newspaper databases. The VU sentences were also used in SWIRT.

A subsequent SRT50 measurement was performed with the speech material from SVT. Since this material is spoken by a male instead of a female speaker and the sentence structure is different, the SRT50 is likely to be different. Only sentences containing true statements were used for the SRT50 measurement.

#### Training and Instruction

A training list of 13 VU sentences was first presented in a quiet environment to allow participants to become familiar with the test and its materials. Participants failing to achieve a word score above 70% in quiet conditions were excluded from subsequent tests. After each sentence, participants were asked to repeat the words they had heard, guessing was allowed.

#### Adaptive Procedure

The presentation level of the sentences was fixed at 70 dB (SPL), and they were presented from a frontal loudspeaker. The sentences were presented in steady-state speech-spectrum noise that matched the speech material, so that different noise was used for the SRT50 measurement with the VU sentences and the SVT material. The noise level was adapted based on the percentage of correctly repeated words from the previous sentence to achieve a word score of 50% correct. An initial SNR value of +2 dB was used. The adaptive procedure used was a stochastic approximation method with an SNR step size 4⋅(50%−Pc(n−1)) , where Pc(n−1) is the percentage of correctly repeated words in the previous sentence (Dingemanse & Goedegebure, 2019a; Robbins & Monro, 1951). A total of 26 sentences were used to determine the SRT50, and the 27th SNR was calculated from the adaptive formula based on the word score of the 26th sentence. The SRT50 is defined as the average over SNRs 5 through 27.

#### Resulting SNR Conditions

The SNR conditions (SRT50 + 4 dB and SRT50 + 8 dB) for SWIRT were based on the SRT50 measurement with the VU sentences, and the SNR conditions for SVT were based on the SRT50 measurement with the SVT material.

### Sentence Verification Test (SVT)

#### Speech Material

True or false Dutch sentences were used, spoken by a native Dutch male speaker, and recorded by Adank and Janse (Adank & Janse, 2009; Janse & Adank, 2012). A total of 100 sentences contained a true statement (e.g., *Tomaten groeien aan planten*, Tomatoes grow on plants) and 100 contained a false or nonsense statement (e.g., *Tomaten hebben sterke tanden*, Tomatoes have strong teeth). False sentences were generated by pairing a subject noun from one sentence with a mismatching predicate from another, creating pairs with either matching or mismatching nouns. For clarity, five sentence pairs that were deemed ambiguously true/false were excluded, resulting in a final set of 95 pairs.

#### Training and Instruction

Prior to the formal test, participants underwent a training session consisting of 10 sentences (five true, five false) to familiarize them with the task. Participants were instructed to determine the veracity of each sentence and indicate their response by pressing a green button for true sentences or a red button for false sentences on a touchscreen as accurately and quickly as possible. Responses given between 0.8 s before and 3.0 s after the end of the sentence were considered valid.

#### Procedure

The sentences were presented at 70 dB (SPL) in steady-state speech-spectrum noise matched to the male speaker's voice in the sentence material. In the main test phase, 30 sentences (15 true, 15 false) were measured per condition. A 3.0-s silence interval separated the end of one sentence from the beginning of the next, allowing participants sufficient time to respond. Instances where no response was given within 3.0 s were recorded as missing responses. The two primary outcomes measured were reaction time (reflecting listening effort) and the proportion of correctly classified sentences (reflecting speech intelligibility).

### Speech Recall Test (SWIRT)

#### Speech Material and List Selection

Dutch VU sentences (Versfeld et al., 2000) ending in two- or three-syllable words were used as stimuli. Lists of seven sentences were created for SWIRT, by extracting the first seven sentences ending in two- or three-syllable words from each VU list of 13 sentences. This kept the sentences from one VU list together, as they were sorted based on phonological content (Versfeld et al., 2000). List 1 was always used for the SRT50 measurement, and the other lists were used for SWIRT to ensure that the sentences selected were different from those used in the SRT50 measurement. Four lists (out of 39) contained less than seven sentences with two- or three-syllable final words and were therefore not used for SWIRT.

#### Training and Instruction

To familiarize participants with the task, a training list of five sentences was presented. Lunner et al. (2020) suggested varying the difficulty of the task to better match individual cognitive abilities. Therefore, the appropriate list length (either five or seven sentences) was determined based on the participants’ ability to recall words during training. If participants were able to recall 0–4 words, SWIRT was conducted with a list length of five sentences. For those who correctly recalled all 5 words, SWIRT was performed with a list length of seven sentences to avoid ceiling effects. Participants were instructed to repeat the last word after each sentence. After the entire list was presented, they were asked to recall as many of the final words as possible, in any order. If the last word was not understood at all, participants were instructed to name and remember another word, so that reduced intelligibility would not affect recall performance.

#### Procedure

Sentences were presented at 70 dB (SPL) in steady-state speech-spectrum noise matched to the female speaker. Five lists were measured in each SNR condition. If a final word was incorrectly repeated, but the incorrectly repeated word was recalled, this was counted as a correct recall. The two outcomes were the proportion of words correctly recalled at the end of the task (reflecting listening effort) and the proportion of words correctly repeated after each sentence (reflecting speech intelligibility).

### Randomization and Test Sessions

The study began with SRT50 measurements to establish SNR values at SRT50 + 4 dB and +8 dB. SWIRT and SVT were then conducted in these SNR conditions with randomly selected test items and lists. To prevent bias, the order of SNR conditions and the sequence of SWIRT and SVT were counterbalanced across participants. A retest session was done for SWIRT and SVT in both SNR conditions approximately one week later with the same participants.

### Setup

Sentences and background noise were presented over a single Genelec 8020 loudspeaker placed 1.13 m in front of the participant at ear level inside a sound booth. The loudspeaker was connected to a Focusrite Scarlett Solo 3rd Gen USB audio interface located outside the sound booth, which was controlled by a PC running TASCAR version 0.222 (Grimm et al., 2019) and MATLAB R2021a. For SWIRT and SRT50 measurements, responses were entered manually by the experimenter in a MATLAB GUI. For SVT, participants touched the corresponding true/false button on an interface on an Android smartphone. A wireless solution was needed because the experimenter and PC were outside of the sound booth. The interface was designed using the TouchOSC app and sent an open sound control (OSC) message to the TASCAR data logging when a button was pressed or released. Button press events were stored in the data logging with a constant low latency of less than 40 ms (SD <23 ms).

### Analyses

#### GLMM Fitting

The primary study variables are the two listening-effort-related measures: the proportion of correctly recalled last words from the sentences after presentation of the entire list in SWIRT and the reaction time when categorizing sentences as true/false in SVT. The secondary study variables are the two speech-intelligibility-related measures: the proportion of correctly repeated last words after presentation of the sentences in SWIRT and the proportion of sentences categorized correctly as true/false in SVT.

To examine the effects of the SNR condition and test/retest condition on the study variables, the data were analyzed using generalized linear mixed-effects models (GLMMs). GLMMs can handle non-normally distributed variables and missing data points. GLMMs are increasingly used in hearing science in recent years (e.g., Lee & Lee, 2022; Prodi et al., 2021), and are believed to be a superior analysis method compared to ANOVA (Jaeger, 2008; Quené & van den Bergh, 2008). GLMMs were fitted in R (version 4.2.2; R_Core_Team, 2023) using the Laplace approximation procedure with the “lme4” package (version 1.1.31; Bates et al., 2015), and described using the “report” package (version 0.5.5; Makowski et al., 2020). The R scripts used for the analysis can be found in the accompanying dataset (Hendrikse et al., 2022).

A GLMM was fitted for each of the study variables. The goal of the GLMM is to achieve the best possible model fit to explain as much variance as possible with as little complexity as possible. To do this, we first specified a model that included all fixed and random effects that could affect the study variable. Fixed effects include the SNR condition and test/retest condition, among other effects. The random effects include a random intercept per participant and per list/item, and random slopes for the SNR condition per participant and per list/item. This is because the psychometric curve may differ between participants and the difficulty may differ between items/lists. In formula form, this looks like: Variable∼SNRcondition+testcondition+other $fixed\;effects+(1_{\;\mathrm{intercept}}+{\mathrm{SNR}\;\mathrm{condition}}_{\mathrm{slope}}{|}_{\mathrm{per}}\;\mathrm{participant},$ $list,\;item{)}_{\mathrm{random}\;\mathrm{effects}}$ . Next, we checked if this model converged. If not, the random effects structure had to be simplified by removing some of the random slopes. The best-fitting model was then determined using the Akaike information criterion (AIC; Akaike, 1973), which is a measure of the relative quality of a statistical model for a given set of data. We iteratively simplified the model by removing one random or fixed effect at a time and recalculating the AIC for the reduced model. The model with the lowest AIC was considered the best fitting model. In addition, interactions were added for the remaining fixed factors to see if this would further improve the model fit, and significant interactions were reported separately.

Standardized coefficients were obtained by refitting the best model to the standardized dataset. The 95% confidence intervals (C.I.s) and *p*-values for the standardized coefficients were calculated using a Wald t- or z-distribution approximation. The standardized coefficient for the SNR condition served as an effect size, assessing the sensitivity of each test method to SNR differences, compared to the standardized coefficient for the test/retest condition, indicating test–retest variability.

#### Ability to Detect SNR Difference Within an Individual

To illustrate what both sensitivity and test–retest variability mean for each test's ability to detect intra-individual differences in SNR, we evaluated how many participants showed a significant intra-individual difference between SNR conditions. We consider an intra-individual difference to be significant if it is larger than the test–retest variability. To obtain a threshold for the test–retest variability, a normal distribution was fitted to the mean test–retest differences per participant. Taking the mean of the absolute values of the 5th and 95th percentiles of the fitted normal distribution yielded a value for which there was only a 5% chance that the test–retest difference was actually larger than this value (the direction of the test–retest difference was irrelevant). This value was used as a threshold to determine the significance of the intra-individual differences. With 18 data points, the fitted normal distribution may not be very accurate, so the percentiles would probably shift with the addition of more data points, potentially resulting in a lower threshold. Given the potential for a lower threshold with more data, we also identified participants who performed better in the SRT50 + 8 dB condition than in the SRT50 + 4 dB condition. The total proportion of participants who did better in the SRT50 + 8 dB condition (regardless of test–retest differences) served as the maximum detection rate for each study variable.

## Results

### Data Distributions

**Figure 2.**
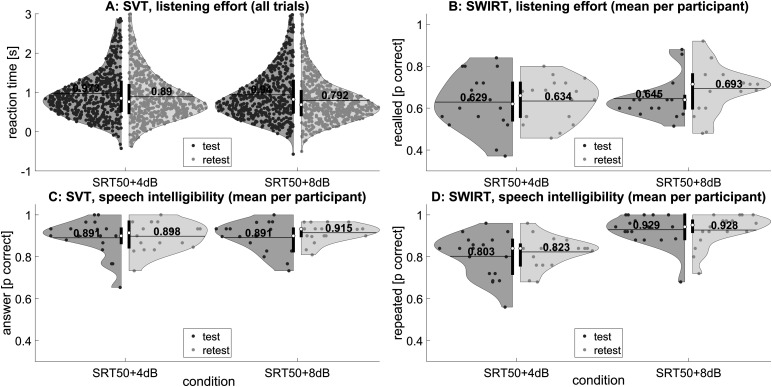
Combined boxplot and density plot of the listening effort (A, B) and speech intelligibility (C, D) measures for SVT (A, C) and SWIRT (B, D) tests. For the reaction time in SVT, data points for all trials are plotted (A) to show the distribution. The other measures have a binomial distribution, so data points for the mean per participant are plotted (B–D). In addition to the data points, the interquartile range (black vertical line), median (white dot), and mean (black horizontal line) are plotted. The mean value is also displayed.

The listening effort and speech intelligibility scores for SVT and SWIRT are shown in Figure 2. For SVT, some trials were missing because participants did not respond within 3 s after the end of the sentence (2.2% of the trials). Binomial distributions were used to fit the proportion of correctly repeated and recalled words in SWIRT and the proportion of correctly categorized sentences in SVT because of the binary nature of the response to each trial (correct or incorrect). As can be seen in Figure 2A, the distribution for reaction times in SVT is positively skewed, that is, not normally distributed. We chose to fit a Gamma distribution to the reaction time data, in accordance with the literature (Baayen & Milin, 2010) and as was done in several other recent studies using reaction time as a measure of listening effort (e.g., Lee & Lee, 2022; Prodi et al., 2021; Visentin et al., 2022). Note that we had to shift the reaction time data by 0.8 s to make them all positive, as the Gamma distribution does not allow negative values. Hence, 0 s corresponded to the earliest possible response that counted, and 0.8 s corresponded to the end of the sentence.

### SRT50 Measurements

**Figure 3.**
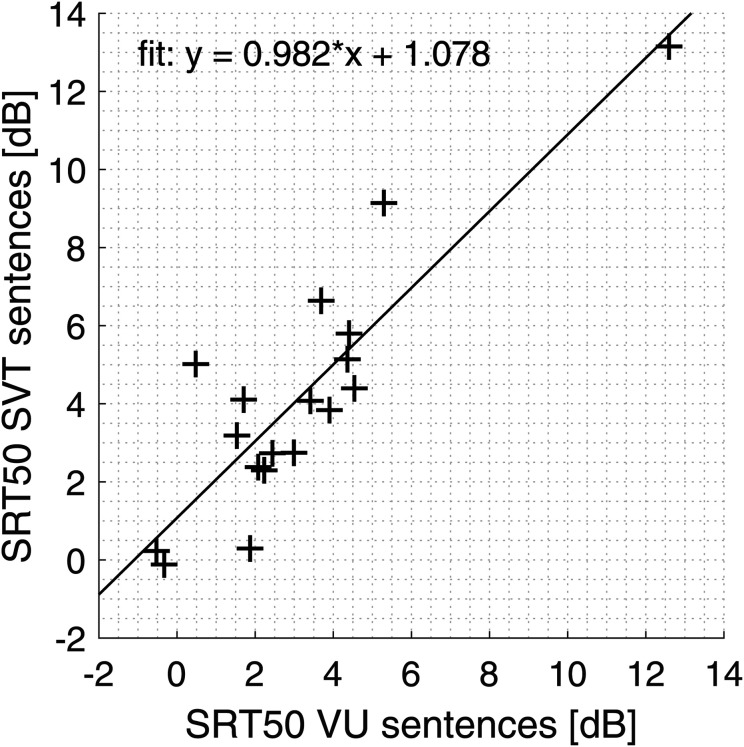
Scatter plot with SRT50 values for all participants for the VU sentences and SVT sentences.

The SRT50 values for the VU sentences (used in SWIRT) and the SVT sentences were compared to check for potential differences in intelligibility between the speech materials. The mean SRT50 values and standard deviations were 3.1 ± 2.9 dB for the VU sentences and 4.2 ± 3.2 dB for the SVT sentences (Figure 3). Since the SRT values with both speech materials are a measure of SRT50, we expected a significant correlation between the SRT50 values, and the Spearman correlation coefficient was calculated to confirm this. As expected, the SRT50 values for the two speech materials were significantly correlated with a Spearman correlation coefficient of 0.74 (*p* < 0.001). This persisted when the most outlying participant with high SRT50 value was removed (Spearman correlation coefficient 0.69, *p* = 0.0027). The SRT50 values were not normally distributed, so a Wilcoxon signed-rank test was performed on the SRT50 values per participant for both speech materials to test for significant differences. The Wilcoxon signed rank test revealed a significant difference between the SRT50 values for the VU and SVT sentences (z-value = −2.72; two-sided *p* = 0.006), indicating slightly better intelligibility for the VU sentences. The mean difference between the SRT50 values for the VU and SVT sentences is 1.1 dB, which is within the 2.4 dB test–retest standard deviation of CI users for the SRT50 values with VU sentences measured by Dingemanse and Goedegebure (2019a). Therefore, the difference between the speech materials is unlikely to be clinically relevant.

### Outliers in Test Items

**Figure 4.**
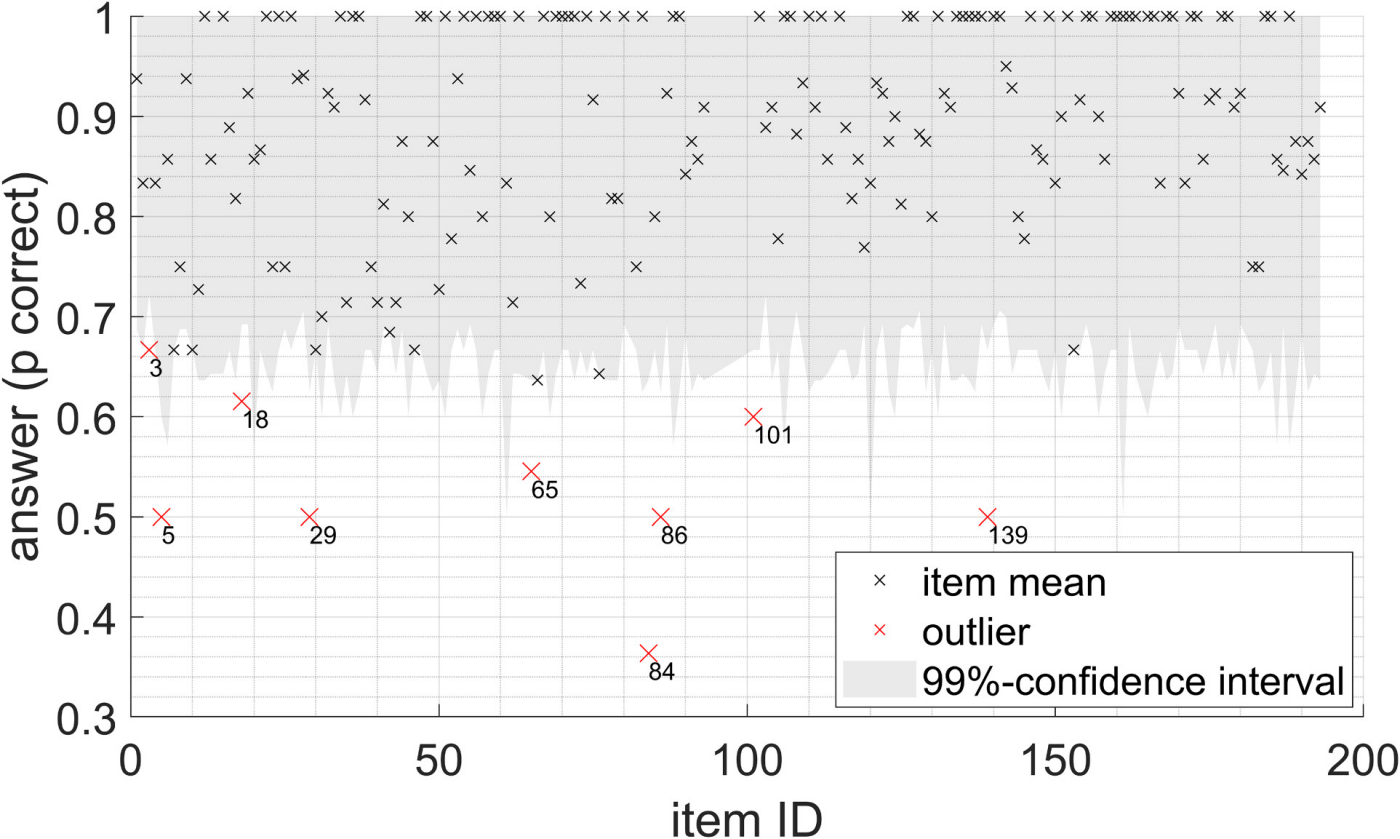
Proportion of trials answered correctly for each item in SVT. For each item, the 99% confidence interval is plotted for a binomial distribution using the fitted chance of success for all data points in the most difficult SNR condition, and the number of trials with that item. Items with means outside this confidence interval are considered outliers.

For both SVT and SWIRT, it was analyzed whether some items/lists could be considered outliers due to statistically different scores for that item/list. Outlier analysis involved fitting a suitable distribution for each study variable to all trials measured in the most difficult condition (SRT50 + 4 dB). The distributions described in the “Data distributions” section were used. Then, for each item/list, the probability that the mean score for that item/list came from the fitted distribution was calculated. If this probability was < 1%, the item/list was considered an outlier. This was the case for several items for the proportion of trials answered correctly in the SVT. The labeled items in Figure 4 (nine in total) are considered outliers. The sentences associated with these item numbers can be found in the accompanying data set. In further analyses, the trials with the items identified as outliers are removed. No outliers were identified for the other three study variables.

### SVT, Listening Effort

#### GLMM Fitting

We fitted a GLMM (Gamma family with log link, estimated using optimx optimizer) to analyze the effect of SNR condition and test/retest condition on the reaction time measured in SVT. The following model was fitted to the data, and after a convergence check and AIC-based model selection, the strikethrough effects were not included in the best-fitting model:

Reaction time ∼ SNR condition + test condition + order + answer + item type + wordscore VU quiet + (1 + SNR condition | participant) + (1 + SNR condition| item)

In this model, “item type” indicates whether the item was true or false, “order” the order of the SNR conditions, “answer” whether the item was correctly categorized, and “word score VU quiet” the word score for the VU training list in quiet. The best model's total explanatory power is substantial (conditional R^2^ = 0.34) and the part related to the fixed effects alone (marginal R^2^) is 0.15. There was no overdispersion. Adding an interaction effect SNRcondition×test×order and the two-way interactions with these fixed effects further improved the model significantly (χ^2^ = 33.87, *p* < 0.001). The three-way interaction effect on reaction time was significant (*p* < 0.001; std. coefficient = 0.23, 95% C.I. [0.14, 0.32]), as were all two-way interactions with these fixed effects.

The parameter estimates (Table 1) indicate a significant effect of SNR condition on reaction time in SVT, and the significant interaction SNRcondition×test×order indicates that there is a learning effect.

**Table 1.** Summary of Fixed Effects for the GLMM of Reaction Time in SVT.

Parameter	Std. coefficient	95% C.I.	*p* value
(Intercept)	0.63	[0.49, 0.78]	<0.001***
SNR condition	0.04	[0.01, 0.08]	0.014*
Test/retest	−0.06	[−0.08, −0.04]	<0.001***
Order	0.16	[−0.03, 0.35]	0.101
Answer	−0.21	[−0.25, −0.17]	<0.001***
Item type	−0.07	[−0.12, −0.01]	0.022*
Word score VU quiet	−0.05	[−0.14, 0.05]	0.342

*<0.05; **<0.01; ***<0.001.

#### Correction for Learning Effect

**Figure 5.**
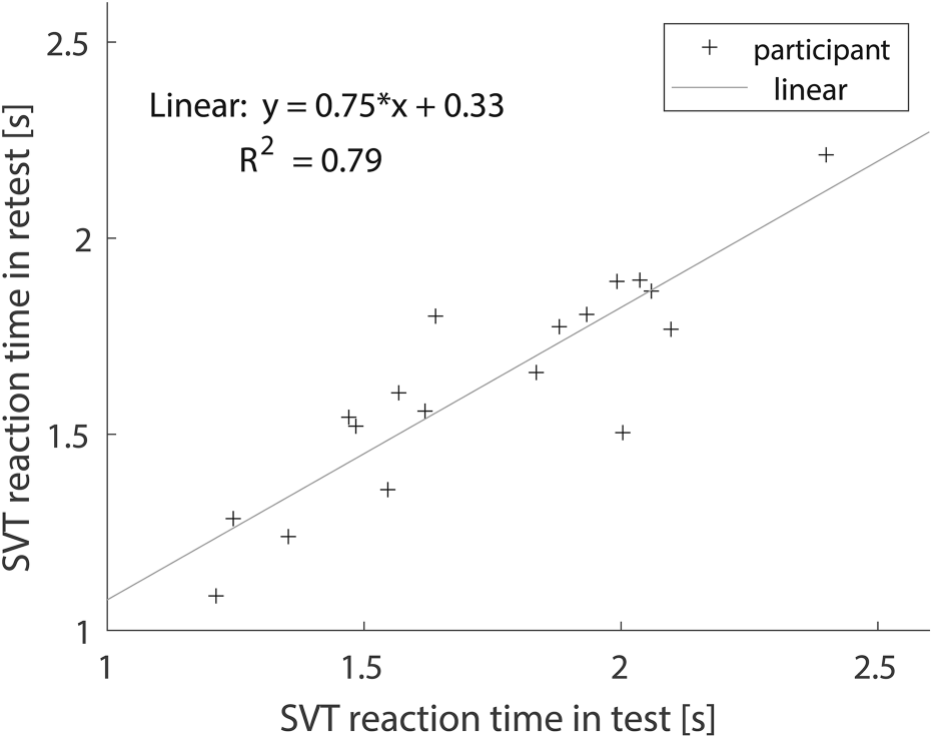
Correlation between the mean SVT reaction time per participant in test and retest.

To visualize this learning effect, the correlation between the mean reaction time per participant in the test and retest is plotted in Figure 5. With an R^2^ value of 0.79 this is a strong correlation, so it is possible to correct for the learning effect to some extent. This means that the standardized coefficient for the test/retest condition is overestimated in the previously fitted GLMM. To obtain a more accurate estimate of the standardized coefficient for the test/retest condition, we corrected the retest data for the learning effect using a linear model fitted to the mean reaction time per participant in the test and retest. The linear model provides an individual correction factor based on the reaction time of a participant in the test, and that individual correction factor was added to the retest data for each participant. The learning effect may also depend on the SNR condition and order of SNR conditions, but fitting separate linear models for these factors did not yield significantly different fits. Therefore, we collapsed across SNR conditions and order of SNR conditions when fitting the linear model. After correcting for the learning effect, we re-fitted the best model, and the resulting parameter estimates are shown in Table 2. As expected, the standardized coefficient for the SNR condition is unchanged, but the standardized coefficient for the test/retest condition is smaller and no longer significant. The three-way interaction SNRcondition×test×order and all two-way interactions with these fixed effects are still significant, because we could not correct for the learning effect in the order of the SNR conditions, as we do not have an estimate of the size of this learning effect. While the standardized coefficient for the SNR condition is now larger than the standardized coefficient for the test/retest condition, their confidence intervals still overlap. This suggests that the effect of the SNR condition on listening effort as measured by the SVT may still be similar in magnitude to the effect of the test/retest condition.

**Table 2.** Summary of Fixed Effects for the GLMM of Reaction Time in SVT, After Correcting for the Learning Effect.

Parameter	Std. coefficient	95% C.I.	*p* value
(Intercept)	1.21	[−0.05, 2.47]	0.061
SNR condition	0.04	[8.64 × 10^−3^, 0.08]	0.014*
Test/retest	−3.03 × 10^−3^	[−0.03, 0.02]	0.788
Order	0.18	[−0.03, 0.39]	0.090
Answer	−0.20	[−0.24, −0.16]	<0.001***
Item type	−0.06	[−0.12, −8.0610 × ^−3^]	0.024*
Word score VU quiet	−0.63	[−1.93, 0.68]	0.345

*<0.05; **<0.01; ***<0.001.

#### Ability to Detect SNR Difference on an Individual Level

The mean intra-individual test–retest differences in reaction time were analyzed, after correction for the learning effect. These mean test–retest differences were normally distributed and fitting a normal distribution to these data points yielded a significance threshold of 211 ms. The SVT reaction time of 2 out of 18 participants was more than 211 ms faster in the SRT50 + 8 dB condition than in the SRT50 + 4 dB condition, indicating that they had a significant intra-individual difference in listening effort between SNR conditions. Twelve out of 18 participants were faster in the SRT50 + 8 dB condition than in the SRT50 + 4 dB condition, which would be the maximum detection rate for the SVT listening effort measure.

### SVT, Speech Intelligibility

#### GLMM Fitting

To analyze the effect of SNR condition on the answer given when categorizing the sentences in SVT, a GLMM (binomial family with logit link, estimated using bobyqa optimizer) was fitted. The following model was fitted to the data, and after a convergence check and AIC-based model selection, the strikethrough effects were not included in the best-fitting model: Answer ∼ SNR condition + test condition + order + item type + wordscore VU quiet + (1 + SNR condition | participant) + (1 + SNR condition | item)

The best model's total explanatory power is moderate (conditional R^2^ = 0.17) and the part related to the fixed effects alone (marginal R^2^) is 0.06. There was no overdispersion. Adding interaction effects did not further improve the model.

The parameter estimates for the best model (Table 3) indicated a significant effect of “word score VU quiet” and item type on the answer given in SVT, but there was no significant effect of SNR condition. This indicates that the SVT speech intelligibility measure was insensitive to the SNR difference. Investigating the effect of item type shows that on average more trials were answered correctly when the sentence was false (93.1%) compared to when the sentence was true (86.8%). Whereas 50% of the trials were true and 50% false, the participants categorized 52.0% of the trials as false, 45.7% as true, and did not respond in 2.3% of the trials. Finally, a better “word score VU quiet” corresponded on average to a higher proportion of correctly answered trials in SVT.

**Table 3.** Summary of Fixed Effects for the GLMM of Answer in SVT.

Parameter	Std. coefficient	95% C.I.	*p* value
(Intercept)	3.03	[2.59, 3.48]	0.293
SNR condition	−0.15	[−0.47, 0.18]	0.384
Test/retest	0.10	[−0.23, 0.42]	0.565
Order	−0.03	[−0.38, 0.32]	0.872
Item type	−0.72	[−1.11, −0.32]	<0.001***
Word score VU quiet	0.34	[0.19, 0.49]	<0.001***

*<0.05; **<0.01; ***<0.001.

#### Ability to Detect SNR Difference on an Individual Level

The mean intra-individual test–retest differences in speech intelligibility measured with SVT were analyzed. These mean test–retest differences were normally distributed and fitting a normal distribution to these data points yielded a significance threshold of 11.1%. Even though there was no significant group effect of the SNR condition, the SVT speech intelligibility score of one participant was more than 11.1% better in the SRT50 + 8 dB condition than in the SRT50 + 4 dB condition, indicating that they had a significant intra-individual difference in speech intelligibility between SNR conditions. Eight out of 18 participants categorized more sentences correctly in the SRT50 + 8 dB condition than in the SRT50 + 4 dB condition, which would be the maximum detection rate for the SVT speech intelligibility measure.

### SWIRT, Listening Effort

#### GLMM Fitting

To analyze the effect of SNR condition on the listening effort measured with SWIRT, a GLMM (binomial family with logit link, estimated using bobyqa optimizer) was fitted. The following model was fitted to the proportion of correctly recalled final words, and after a convergence check and AIC-based model selection, the strikethrough effects were not included in the best-fitting model: Recalled ∼ SNR condition + test condition + order + position in list + word type +wordscore VU quiet + (1 + SNR condition | participant) + (1 + SNR condition | list)

In this model, “position in list” indicates the position of the sentence in the list, and “word type” whether the word type was a verb, noun, adjective, or other. The “position in list” was included, because it is known that the last word from the final sentence in the list is more easily recalled than the last words from the first and middle sentences (Lunner et al., 2016). The “word type” was included, because some participants mentioned that the verbs were more alike and more difficult to remember and we wanted to investigate this. The best model's total explanatory power is moderate (conditional R^2^ = 0.22) and the part related to the fixed effects alone (marginal R^2^) is 0.19. There was no overdispersion. Adding interaction effects did not further improve the model.

The parameter estimates for the best model (Table 4) indicated a significant effect of SNR condition and on the proportion of words recalled correctly. When looking at the mean proportions of words recalled correctly per participant, we see that participants recalled on average 3.8% more words correctly in the SRT50 + 8 dB condition than in the SRT50 + 4 dB condition. The mean difference in proportion of words recalled correctly per participant between test and retest is lower, on average 2.7%. The standardized coefficients for the SNR condition and test/retest condition are similar in size (within each other's 95% C.I.), which indicates that the effect of the SNR condition on the listening effort measured with SWIRT was similar in size compared to the effect of test/retest. Moreover, a significant effect of “position in list” was found, because the last word in the list was significantly more often recalled (92.2%) than the first (64.4%) and middle (57.0%) words. No significant effect of “word type” was found, although some *p*-values are close to 0.05.

**Table 4.** Summary of Fixed Effects for the GLMM of “Recalled” in SWIRT.

Parameter		Std. coefficient	95% C.I.	*p* value
(Intercept)		0.39	[−0.13, 0.90]	0.281
SNR condition		−0.20	[−0.39, 0.00]	0.049*
Test/retest		0.13	[−0.07, 0.33]	0.200
Order		0.006	[−0.43, 0.44]	0.980
Position in list (vs. first)	Middle	−0.33	[−0.58, −0.08]	0.010*
	Last	1.92	[1.47, 2.37]	<0.001***
Word type (vs. adjective)	Noun	0.40	[0.00, 0.80]	0.053
	Other	−0.05	[−0.51, 0.41]	0.830
	Verb	0.29	[−0.12, 0.70]	0.160
Word score VU quiet		0.15	[−0.06, 0.36]	0.155

*<0.05; **<0.01; ***<0.001.

#### Ability to Detect SNR Difference on an Individual Level

The mean intra-individual test–retest differences in listening effort measured with SWIRT were analyzed. These mean test–retest differences were normally distributed and fitting a normal distribution to these data points yielded a significance threshold of 14.4%. One participant recalled more than 14.4% more words in the SRT50 + 8 dB condition than in the SRT50 + 4 dB condition, indicating that the participant had a significant intra-individual difference in listening effort between SNR conditions. Twelve out of 18 participants recalled more words in the SRT50 + 8 dB condition than in the SRT50 + 4 dB condition, which would be the maximum detection rate for the SWIRT listening effort measure.

### SWIRT, Speech Intelligibility

#### GLMM Fitting

A GLMM (binomial family with logit link, estimated using bobyqa optimizer) was fitted to analyze the effect of the SNR condition on the repeated sentence-final words in SWIRT. The following model was fitted to the proportion of correctly repeated final words, and after a convergence check and AIC-based model selection, the strikethrough effects were not included in the best-fitting model: Repeated ∼ SNR condition + test condition + order + position in list + word type + wordscore VU quiet + (1 + SNR condition | participant) + (1 + SNR condition | list)

The best model's total explanatory power is moderate (conditional R^2^ = 0.20) and the part related to the fixed effects alone (marginal R^2^) is 0.12. There was no overdispersion. Adding an interaction effect SNRcondition×wordscoreVUquiet further improved the model significantly (χ^2^ = 6.90, *p* = 0.009), and the interaction effect on the proportion of words repeated correctly was significant (*p* = 0.008; std. coefficient = −0.34, 95% C.I. [−0.59, −0.09]).

The final fit indicated a significant effect of SNR condition (Table 5). When looking at the mean proportions of words repeated correctly per participant, we see that participants repeated on average 11.6% more words correctly in the SRT50 + 8 dB condition than in the SRT50 + 4 dB condition. The mean difference in proportion of words repeated correctly per participant between test and retest was lower, on average 1.0%. The magnitude of the standardized coefficient for SNR condition is much larger than that of the coefficient for test/retest condition. This indicates that the effect of the SNR condition on the speech intelligibility measured with SWIRT was larger than the effect of test/retest. In addition, a significant effect of “word score VU quiet” was found (Table 5). A better “word score VU quiet” resulted on average in a higher proportion of correctly repeated last words, and the interaction showed that the difference in proportion of correctly repeated last words between SNR conditions was larger for participants with a better “word score VU quiet.”

**Table 5.** Summary of Fixed Effects for the GLMM of “Repeated” in SWIRT.

Parameter		Std. coefficient	95% C.I.	*p* value
(Intercept)		2.97	[2.22, 3.72]	0.956
SNR condition		−1.23	[−1.54, −0.92]	<0.001***
Test/retest		0.08	[−0.20, 0.36]	0.565
Order		−0.16	[−0.59, 0.27]	0.474
Position in list (vs. first)	Middle	−0.25	[−0.63, 0.12]	0.188
	Last	−0.08	[−0.56, 0.39]	0.733
Word type (vs. adjective)	Noun	−0.14	[−0.73, 0.45]	0.636
	Other	−0.02	[−0.71, 0.67]	0.951
	Verb	0.26	[−0.34, 0.87]	0.394
Word score VU quiet		0.25	[0.05, 0.45]	0.012*

*<0.05; **<0.01; ***<0.001.

#### Ability to Detect SNR Difference on an Individual Level

The mean intra-individual test–retest differences in speech intelligibility measured with SWIRT were analyzed. These mean test–retest differences were normally distributed and fitting a normal distribution to these data points yielded a significance threshold of 11.5%. Eight out of 18 participants repeated more than 11.5% more words in the SRT50 + 8 dB condition than in the SRT50 + 4 dB condition, indicating that they had a significant intra-individual difference in speech intelligibility between SNR conditions. Seventeen out of 18 participants repeated more words in the SRT50 + 8 dB condition than in the SRT50 + 4 dB condition, which would be the maximum detection rate for the SWIRT speech intelligibility measure.

## Discussion

This study investigated the feasibility of measuring listening effort differences due to different input signal-to-noise ratios (SNRs) in CI users using a sentence verification test (SVT) and a sentence-final word identification and recall test (SWIRT), with the ultimate goal of evaluating and comparing different hearing-device settings in the clinic. In addition, the study investigated whether listening effort measures would be more appropriate than speech intelligibility measures for quantifying the effect of an SNR difference when speech intelligibility is near maximum.

### Main findings

Both SVT and SWIRT measured a significant difference in listening effort between the two SNR conditions, so it is feasible to measure listening effort in CI users at group level using these two behavioral tests. For the listening effort measures of both SVT and SWIRT, the standardized coefficients of the GLMM for SNR condition and test/retest had overlapping 95% C.I.s, even after attempting to correct for the learning effect in SVT. This indicates that the listening effort measures of SVT and SWIRT could not reliably distinguish effects of SNR condition from variability between test sessions. Therefore, a difference in listening effort of this magnitude cannot be reliably measured within an individual with SVT and SWIRT as implemented in the current study.

The inability of SVT and SWIRT to detect intra-individual listening effort differences was further illustrated with an additional analysis. SVT was only able to detect a significant intra-individual difference in listening effort between the SNR conditions within two participants, and SWIRT only within one of the 18 participants. With more data, the estimate of the test–retest variability would become more precise and the significance threshold for intra-individual listening effort differences could shift. However, for both tests only 12 out of 18 participants showed an effect in the correct direction, indicating more listening effort in the more difficult SRT50 + 4 dB condition. Therefore, the maximum detection rate of the listening effort tests for intra-individual differences was 67% for the implementations used in this study, which is a poor performance (near chance). The SVT measure of speech intelligibility did not detect a significant difference between SNR conditions. The SWIRT measure of speech intelligibility performed much better, with a significant difference detected within eight participants and a maximum detection rate of 94% (17 out of 18).

The effect of the SNR condition on the SWIRT speech intelligibility measure was greater than the test–retest difference for this measure and greater than the effect on the listening effort measures. Thus, contrary to our expectations, speech intelligibility measures are better able to discriminate between SNR conditions in adult CI users than the listening effort measures of SVT and SWIRT in their current implementation.

### Intelligibility Differences Between SNR Conditions

The choice of SNR conditions relative to the individual SRT50 values in the current study also means that the difference in speech intelligibility between the two conditions will vary between participants. The difference in listening effort between the two conditions may therefore also vary between participants. However, as the conditions were chosen in the range where the psychometric curve flattens, this variation in intelligibility difference is minimal: the difference in the proportion of correct words between the conditions would be at least 0.04 and at most 0.10 (Figure 1). Moreover, this choice of conditions was important for the study design. The SNR conditions of SRT50 + 4 dB and SRT50 + 8 dB were chosen to be at a relatively high level of intelligibility, with an SNR difference that reflected a reduction in SNR that could be achieved by an effective noise reduction algorithm. Because another study with CI users did not measure listening effort differences between SNRs at which speech perception scores were maximal (Perreau et al., 2017), a difference in intelligibility between conditions was necessary to ensure a difference in listening effort in the current study. The SNR conditions were chosen to be at a relatively high level of intelligibility, because in this SNR region speech intelligibility measures are less sensitive than around SRT50, and listening effort measures are believed to be more informative.

### Intelligibility Differences Between Speech Materials

To allow a fair comparison between the SVT and SWIRT test procedures, it was necessary to ensure that the SVT and SWIRT were measured at the same level of speech intelligibility performance. Therefore, the SRT50 was measured for both the VU sentences used in SWIRT and the speech material used in SVT to account for potential differences in intelligibility between the speech materials. We also analyzed whether there was actually a difference in intelligibility between the speech materials. As expected, the SRT50 values for the different speech materials were significantly correlated. The SRT50 values show that there was a significant difference of 1.1 dB between the speech materials and that the speech material used in SVT was slightly more difficult to understand. This effect is unlikely to be clinically relevant, because it is within the test–retest standard deviation of SRT50 values measured with the VU sentences in CI users. The effect of the speech material could be related to the different structure, as the statements used in SVT have less context than the sentences used in SWIRT. Alternatively, it could be related to the fact that the SVT material has a male speaker instead of a female speaker, due to their different frequency ranges and the resulting differences in audibility for people with hearing loss. This is a small effect but should be taken into account when comparing listening effort test methods with different speech materials.

### Comparison to Literature

Like previous studies using the SVT and SWIRT (Lunner et al., 2016; Ng et al., 2013; Pals et al., 2020), the current study found group-level effects of the two test conditions on listening effort measured with the SVT and SWIRT. Neither of these previous studies evaluated test–retest variability and its effect on the ability of the tests to detect intra-individual differences. The current study evaluated the ability of both tests to detect intra-individual differences in listening effort in CI users, with disappointing results. In the introduction, we wrote that if the SVT and SWIRT were found to be effective for CI users, they would probably be suitable for hearing-aid users as well. Given our findings of ineffectiveness in detecting intra-individual differences in CI users, we sought to extrapolate the implications for other populations. Consequently, we present an overview of studies that have compared listening effort in CI users with other populations.

Most CI users have a reduced ability to fully understand speech, even in quiet. Several years ago, Ohlenforst et al. (2017) concluded from their literature review on listening effort that there is scientific evidence from physiological methods suggesting that hearing impairment leads to increased listening effort. Dingemanse and Goedegebure (2022) showed that pupillometry measurements indicated only a slight reduction in listening effort with increasing SNR (for 50%, 70%, and near maximum speech intelligibility) in CI users. They compared their results with those of Zekveld et al. (2010, 2011) and found that the reduction in listening effort for CI users was substantially smaller compared to the reduction found for NH listeners. The results of Perreau et al. (2017) also showed that listening effort was reduced to a greater extent in NH listeners compared to CI users with increasing SNR, using a dual-task paradigm with a reaction time measure. However, in a recent study by Abdel-Latif and Meister (2022), neither subjective (measured at 50% and 80% speech intelligibility) nor behavioral measures (measured at 80% speech intelligibility) indicated a difference in listening effort between NH listeners and CI users. Abdel-Latif and Meister explain their different results by the underlying differences in psychometric functions of NH and CI users, which were not compensated by Perreau et al. (2017) because SNR conditions were used instead of speech intelligibility performance conditions. If it is true that listening effort follows speech intelligibility performance, it could also be that the minimum listening effort of CI users remains higher than that of NH listeners when CI users are unable to achieve 100% speech intelligibility, or even under ideal listening conditions when they do achieve 100% speech intelligibility. This could explain the difference between the results of Abdel-Latif and Meister and Dingemanse and Goedegebure (2022), who included a near-maximum speech intelligibility condition where such a difference in minimum listening effort would emerge. The smaller reduction in listening effort per SNR increment and the higher minimum listening effort for CI users would make it more difficult to measure differences in listening effort for CI users compared to NH listeners. For hearing-aid users, comparisons of listening effort with NH listeners have been inconclusive (Ohlenforst et al., 2017), and no studies were found that directly compared listening effort in CI users and hearing-aid users. Therefore, we do not know whether it is more difficult to measure differences in listening effort for CI users compared to hearing-aid users. Thus, although the SVT and SWIRT are not able to detect intra-individual differences in listening effort in CI users, they might still be able to detect intra-individual differences in listening effort in NH listeners and hearing-aid users.

### Points of Improvement for Test Procedures

The implementations of SVT and SWIRT used in this study cannot reliably detect intra-individual differences in listening effort. However, there are several ways to improve the test–retest reliability of the tests, which would also improve their ability to detect intra-individual differences in listening effort.

#### Improving the SVT

##### Correcting for Learning Effects

The significant interaction SNRcondition×test×order indicates that there is a learning effect for the SVT listening effort measure. Pals et al. (2020) also found a decrease in reaction time with SVT for consecutive test blocks in CI users. A training list of 10 sentences (5 true, 5 false) was used prior to testing, but since the learning effect was still present after 60 trials, training would not be sufficient to remove it. We corrected the retest data for the learning effect, but the learning effect in the order of the SNR conditions could not be corrected. After correction, the test–retest reliability of the SVT listening effort measure improved, but not enough to reliably detect intra-individual differences in listening effort. With more data on how this learning effect behaves, a better correction would be possible, also for the learning effect between two conditions measured within a session. A better correction for the learning effect could further improve the test–retest reliability. However, there is likely to be individual variability in learning, so it is unlikely that it can be completely eliminated. Interleaving conditions is advisable when using the SVT in future studies. This would remove the effect of the order of test conditions, which is better for analyzing intra-individual differences. However, it would also increase the variability of scores within one condition, making it more difficult to detect differences between conditions.

##### Optimizing Speech Material

The Dutch SVT material has been used previously in other studies (Adank & Janse, 2009; Janse & Adank, 2012; Pals et al., 2020), but a thorough analysis of the test material was lacking, so an analysis to identify potential outliers was also conducted. For some SVT items, the proportion of trials answered correctly was significantly lower. This could be because a fact was not as widely known as assumed (e.g., “parchment is made of leather”), or perhaps the sentence was less clearly pronounced than others. Since the SVT was not intended to be a speech intelligibility test, the intelligibility of the sentences was not controlled for. Removing outliers in the SVT items might improve the test–retest reliability of the tests somewhat.

##### Improving Equipment Latency

The TouchOSC smartphone interface used to record the participants’ responses in the SVT was chosen because it was wireless and could be implemented on equipment we already had available. However, there are devices available for which the standard deviation of the latency (<4 ms; Plant & Turner, 2009) is smaller than that of the smartphone interface (<23 ms). Therefore, the use of different equipment might also slightly improve the test–retest reliability.

##### Increasing the Number of Trials

The current study used 30 trials (15 true, 15 false) per condition for the SVT, which is the same number of trials as used by Pals et al. (2020). The median measurement time for 30 trials was 5 min and 29 s. A slightly longer measurement time would probably still be feasible in a clinical setting, so it would be possible to increase the number of trials. More trials per condition usually improve test–retest reliability, but there would also be a greater learning effect between the first and last trial of the condition, which would counteract this effect. Thus, the optimal number of trials for the SVT needs to be investigated.

The analysis also showed that the false sentences were categorized correctly more often, but more slowly, than the true sentences. In addition, the sentences were categorized as “false” more often. A possible explanation is that if one or more words in the sentence are misunderstood, it is likely to be categorized as “false.” This creates a slight bias toward the “false” category and a higher chance of correctly categorizing false sentences. Therefore, it is important to use the same ratio of true to false sentences for different test conditions.

#### Improving the SWIRT

##### Optimizing Speech Material

The SWIRT was administered for the first time with Dutch sentences. Therefore, a thorough analysis of potential outliers in the test lists and sentences was performed to gain insight into the test quality. No outliers were found in the SWIRT lists. However, some words appeared in multiple lists (e.g., “open”). Whether the word was a noun, verb, adjective, or other word had no significant effect on listening effort as measured by SWIRT, but had a medium effect size that could become significant with more data points. Therefore, future studies should further investigate the effect of word type. Perhaps the sentences in the SWIRT lists that were created should be rearranged into new lists that are explicitly balanced for the memorability and occurrence of the final words. This may improve test–retest reliability.

##### Increasing Number of Lists and Adjusting List Length

In the current study, five lists per condition were measured for the SWIRT, as was done by Lunner et al. (2016). The median measurement time for five lists was 8 min and 42 s. We do not believe that a longer measurement time would be feasible in a clinical setting, especially since the task is also quite demanding. Therefore, increasing the number of lists to improve test–retest reliability is probably not feasible.

An adaptive list length of either five or seven sentences was used to avoid ceiling effects. Four participants used a seven-sentence list, and the rest used five sentences. This procedure seemed to be effective in preventing ceiling effects, as the highest mean percentage of correctly recalled words per participant was 0.90 and the lowest was 0.43.

### Other Test Methods

In both tests included in the current study, the secondary task was auditory-related. A dual-task paradigm in which the secondary task is not auditory-related, such as the detection of a visual stimulus, as in the paradigm used by Desjardins and Doherty (2014), was not included in the current study. It is still unclear which type of secondary task is most appropriate (Gagne et al., 2017), so it would be good to compare listening effort tests with other types of secondary tasks in future research.

### Clinical Implications

For clinical use, it is important that the tests allow conclusions based on individual measurements rather than group data, so that the tests can be used to find the optimal hearing-device setting for an individual. The results show that if different hearing-device settings cause a difference in both speech intelligibility and listening effort, speech intelligibility tests are more likely to find the better CI setting for an individual than the SVT and SWIRT listening effort measures for the test implementations used in this study, even at a relatively high speech intelligibility.

Of course, different hearing-device settings may cause differences that go beyond intelligibility, such as differences in sound quality, which can also affect listening effort. For example, Pals et al. (2020) have shown that the number of active electrodes affects listening effort in CI users. So, even though the SWIRT speech intelligibility measure was better able to differentiate between the SRT50 + 4 dB and SRT50 + 8 dB conditions than the listening effort measures, this may not be the case when comparing different hearing-device settings at a higher intelligibility level. We do think that the difference in listening effort between the conditions in the current study would be similar to the listening effort difference that can be expected from different hearing-device settings, since the 4 dB difference between conditions reflects the SNR reduction that an effective noise reduction algorithm could achieve (Brons et al., 2013). Therefore, the implementations of SVT and SWIRT used in the current study would not be able to reliably detect within-individual differences in listening effort for different CI settings.

## Conclusion

Both the SVT and the SWIRT were able to detect a significant difference in listening effort between the SNR conditions on group level. However, neither SVT nor SWIRT could reliably distinguish effects of SNR condition from test–retest variability, so the SVT and SWIRT test procedures used in this study were unable to reliably detect intra-individual differences in listening effort. SWIRT also detected a significant difference in speech intelligibility between the SNR conditions. The effect of SNR condition on the SWIRT speech intelligibility measure was greater than the test–retest difference for this measure and greater than the effect on the listening effort measures. In terms of clinical feasibility, this means that a speech intelligibility test is much more likely to find optimal CI settings for an individual than the listening effort tests used in this study, even when speech intelligibility is near maximum. In case two CI settings only result in a difference in listening effort but not in speech intelligibility, it is also unlikely that this difference is large enough to be reliably detected within an individual by the SVT and SWIRT test procedures used in this study. However, SVT and SWIRT are appropriate for research purposes with CI users when group data can be analyzed. Further research is needed to determine whether the SVT and SWIRT test procedures can be improved sufficiently to make them suitable for clinical use. The results emphasize the need to assess test–retest variability and to make direct comparisons with speech intelligibility measures in future studies evaluating listening effort test procedures.

## References

[bibr1-23312165241240572] Abdel-Latif K. H. A. Meister H. (2022). Speech recognition and listening effort in cochlear implant recipients and normal-hearing listeners. Frontiers in Neuroscience, 15:725412, 1–13. 10.3389/fnins.2021.725412 PMC8867819 35221883

[bibr2-23312165241240572] Adank P. Janse E. (2009). Perceptual learning of time-compressed and natural fast speech. Journal of the Acoustical Society of America, 126(5), 2649–2659. 10.1121/1.3216914 19894842

[bibr3-23312165241240572] Akaike H. (1973). *Information theory and an extension of the maximum likelihood principle.* Paper presented at the 2nd International Symposium on Information Theory, 1973.

[bibr4-23312165241240572] Baayen R. H. Milin P. (2010). Analyzing reaction times. International Journal of Psychological Research, 3(2), 12–28. 10.21500/20112084.807

[bibr5-23312165241240572] Baddeley A. D. Emslie H. Nimmo-Smith I. (1992). The speed and capacity of language-processing test. Thames Valley Test Company.

[bibr6-23312165241240572] Bates D. Mächler M. Bolker B. Walker S. (2015). Fitting linear mixed-effects models using lme4. Journal of Statistical Software, 67(1), 1–48. 10.18637/jss.v067.i01

[bibr7-23312165241240572] Brons I. Houben R. Dreschler W. A. (2013). Perceptual effects of noise reduction with respect to personal preference, speech intelligibility, and listening effort. Ear and Hearing, 34(1), 29–41. 10.1097/AUD.0b013e31825f299f 22874643

[bibr8-23312165241240572] Chang H.-P. Ho C.-Y. Chou P. (2009). The factors associated with a self-perceived hearing handicap in elderly people with hearing impairment—results from a community-based study. Ear and Hearing, 30(5), 576–583. 10.1097/AUD.0b013e3181ac127a 19633566

[bibr9-23312165241240572] Desjardins J. L. Doherty K. A. (2014). The effect of hearing aid noise reduction on listening effort in hearing-impaired adults. Ear and Hearing, 35(6), 600–610. 10.1097/AUD.0000000000000028 24622352

[bibr10-23312165241240572] Dingemanse J. G. Goedegebure A. (2019a). Efficient adaptive speech reception threshold measurements using stochastic approximation algorithms. Trends in Hearing, 23, 1–17. 10.1177/2331216520919199 PMC7238302 32425135

[bibr11-23312165241240572] Dingemanse J. G. Goedegebure A. (2019b). The important role of contextual information in speech perception in cochlear implant users and its consequences in speech tests. Trends in Hearing, 23, 1–16. 10.1177/2331216519838672 PMC6472157 30991904

[bibr12-23312165241240572] Dingemanse J. G. Goedegebure A. (2020). The relation of hearing-specific patient-reported outcome measures with speech perception measures and acceptable noise levels in cochlear implant users. International Journal of Audiology, 59(6), 416–426. 10.1080/14992027.2020.1727033 32091274

[bibr13-23312165241240572] Dingemanse J. G. Goedegebure A. (2022). Listening effort in cochlear implant users: The effect of speech intelligibility, noise reduction processing, and working memory capacity on the pupil dilation response. Journal of Speech, Language, and Hearing Research, 65(1), 392–404. 10.1044/2021_JSLHR-21-00230 34898265

[bibr14-23312165241240572] Francis A. L. Love J. (2020). Listening effort: Are we measuring cognition or affect, or both? Wiley Interdisciplinary Reviews: Cognitive Science, 11(1), e1514. 10.1002/wcs.1514 31381275

[bibr15-23312165241240572] Gagne J.-P. Besser J. Lemke U. (2017). Behavioral assessment of listening effort using a dual-task paradigm: A review. Trends in Hearing, 21. 10.1177/2331216516687287 PMC5308443 28091178

[bibr16-23312165241240572] Granberg S. Pronk M. Swanepoel D. W. Kramer S. E. Hagsten H. Hjaldahl J. Danermark B. (2014). The ICF core sets for hearing loss project: Functioning and disability from the patient perspective. International Journal of Audiology, 53(11), 777–786. 10.3109/14992027.2014.938370 25311099

[bibr17-23312165241240572] Grimm G. Luberadzka J. Hohmann V. (2019). A toolbox for rendering virtual acoustic environments in the context of audiology. Acta Acustica United with Acustica, 105(3), 566–578. 10.3813/AAA.919337

[bibr18-23312165241240572] Hendrikse M. Dingemanse G. Goedegebure A. (2022). Data set of two dual-task paradigms to measure listening effort in cochlear implant users. 10.5281/zenodo.6973725 PMC11055488 38676325

[bibr19-23312165241240572] Jaeger T. F. (2008). Categorical data analysis: Away from ANOVAs (transformation or not) and towards logit mixed models. Journal of Memory and Language, 59(4), 434–446. 10.1016/j.jml.2007.11.007 19884961 PMC2613284

[bibr20-23312165241240572] Janse E. Adank P. (2012). Predicting foreign-accent adaptation in older adults. Q J Exp Psychol (Hove), 65(8), 1563–1585. 10.1080/17470218.2012.658822 22530648

[bibr21-23312165241240572] Kahneman D. (1973). Attention and effort (Vol. 1063). Citeseer.

[bibr22-23312165241240572] Lee S. J. Lee S. (2022). Clinical utility of response time in speech audiometry in elderly with mild cognitive impairment. International Journal of Audiology, 62(5), 1–6. 10.1080/14992027.2022.2047234 35289698

[bibr23-23312165241240572] Lunner T. Alickovic E. Graversen C. Ng E. H. N. Wendt D. Keidser G. (2020). Three new outcome measures that tap into cognitive processes required for real-life communication. Ear and Hearing, 41(Suppl 1), 39S–47S. 10.1097/AUD.0000000000000941 33105258 PMC7676869

[bibr24-23312165241240572] Lunner T. Rudner M. Rosenbom T. Agren J. Ng E. H. (2016). Using speech recall in hearing aid fitting and outcome evaluation under ecological test conditions. Ear and Hearing, 37(Suppl 1), 145S–154S. 10.1097/AUD.0000000000000294 27355764

[bibr25-23312165241240572] Makowski D. Ben-Shachar M. Patil I. Lüdecke D. (2020). Automated results reporting as a practical tool to improve reproducibility and methodological best practices adoption. *CRAN.* https://github.com/easystats/report

[bibr26-23312165241240572] May J. Alcock K. J. Robinson L. Mwita C. (2001). A computerized test of speed of language comprehension unconfounded by literacy. Applied Cognitive Psychology: The Official Journal of the Society for Applied Research in Memory and Cognition, 15(4), 433–443. 10.1002/acp.715

[bibr27-23312165241240572] Ng E. H. Rudner M. Lunner T. Pedersen M. S. Ronnberg J. (2013). Effects of noise and working memory capacity on memory processing of speech for hearing-aid users. International Journal of Audiology, 52(7), 433–441. 10.3109/14992027.2013.776181 23550584

[bibr28-23312165241240572] Ng E. H. Rudner M. Lunner T. Ronnberg J. (2015). Noise reduction improves memory for target language speech in competing native but not foreign language speech. Ear and Hearing, 36(1), 82–91. 10.1097/AUD.0000000000000080 25166628

[bibr29-23312165241240572] Ohlenforst B. Zekveld A. A. Jansma E. P. Wang Y. Naylor G. Lorens A. Kramer S. E. (2017). Effects of hearing impairment and hearing aid amplification on listening effort: A systematic review. Ear and Hearing, 38(3), 267–281. 10.1097/AUD.0000000000000396 28234670 PMC5405775

[bibr30-23312165241240572] Pals C. Sarampalis A. Beynon A. Stainsby T. Baskent D. (2020). Effect of spectral channels on speech recognition, comprehension, and listening effort in cochlear-implant users. Trends in Hearing, 24, 2331216520904617. 10.1177/2331216520904617 32189585 PMC7082863

[bibr31-23312165241240572] Perreau A. E. Wu Y.-H. Tatge B. Irwin D. Corts D. (2017). Listening effort measured in adults with normal hearing and cochlear implants. Journal of the American Academy of Audiology, 28(8), 685–697. 10.3766/jaaa.16014 28906240 PMC6135240

[bibr32-23312165241240572] Pichora-Fuller M. K. Kramer S. E. Eckert M. A. Edwards B. Hornsby B. W. Y. Humes L. E. Mackersie C. L. (2016). Hearing impairment and cognitive energy: The framework for understanding effortful listening (FUEL). Ear and Hearing, 37, 5S–27S. 10.1097/AUD.0000000000000312 27355771

[bibr33-23312165241240572] Plant R. R. Turner G. (2009). Millisecond precision psychological research in a world of commodity computers: New hardware, new problems? Behavior Research Methods, 41(3), 598–614. 10.3758/BRM.41.3.598 19587169

[bibr34-23312165241240572] Prodi N. Visentin C. Borella E. Mammarella I. C. Di Domenico A. (2021). Using speech comprehension to qualify communication in classrooms: Influence of listening condition, task complexity and students’ age and linguistic abilities. Applied Acoustics, 182, 108239. 10.1016/j.apacoust.2021.108239

[bibr35-23312165241240572] Quené H. van den Bergh H. (2008). Examples of mixed-effects modeling with crossed random effects and with binomial data. Journal of Memory and Language, 59(4), 413–425. 10.1016/j.jml.2008.02.002

[bibr36-23312165241240572] R_Core_Team (2023). R: A language and environment for statistical computing. R Foundation for Statistical Computing. https://www.r-project.org/

[bibr37-23312165241240572] Robbins H. Monro S. (1951). A stochastic approximation method. The Annals of Mathematical Statistics, 22(3), 400–407. 10.1214/aoms/1177729586

[bibr38-23312165241240572] Shinn-Cunningham B. G. Best V. (2008). Selective attention in normal and impaired hearing. Trends in Amplification, 12(4), 283–299. 10.1177/1084713808325306 18974202 PMC2700845

[bibr39-23312165241240572] Smeds K. Dahlquist M. Larsson J. Herrlin P. Wolters F. (2019, September 9–13). *LEAP, a new laboratory test for evaluating auditory preference.* Paper presented at the 23rd International Congress on Acoustics, Aachen, Germany.

[bibr40-23312165241240572] Timmer B. H. B. Hickson L. Launer S. (2018). Do hearing aids address real-world hearing difficulties for adults with mild hearing impairment? Results from a pilot study using ecological momentary assessment. Trends in Hearing, 22, 1–15. 10.1177/2331216518783608 PMC6048604 29956590

[bibr41-23312165241240572] Versfeld N. J. Daalder L. Festen J. M. Houtgast T. (2000). Method for the selection of sentence materials for efficient measurement of the speech reception threshold. Journal of the Acoustical Society of America, 107(3), 1671–1684. 10.1121/1.428451 10738820

[bibr42-23312165241240572] Visentin C. Volzolgher C. Pellegatti M. Potente P. Pavani F. Prodi N. (2022). A comparison of simultaneously-obtained measures of listening effort: Pupil dilation, verbal response time and self-rating. International Journal of Audiology, 61(7), 561–573. 10.1080/14992027.2021.1921290 34634214

[bibr43-23312165241240572] Zekveld A. A. Kramer S. E. Festen J. M. (2010). Pupil response as an indication of effortful listening: The influence of sentence intelligibility. Ear and Hearing, 31(4), 480–490. 10.1097/AUD.0b013e3181d4f251 20588118

[bibr44-23312165241240572] Zekveld A. A. Kramer S. E. Festen J. M. (2011). Cognitive load during speech perception in noise: The influence of age, hearing loss, and cognition on the pupil response. Ear and Hearing, 32(4), 498–510. 10.1097/AUD.0b013e31820512bb 21233711

